# Multi-Objective Task-Aware Offloading and Scheduling Framework for Internet of Things Logistics

**DOI:** 10.3390/s24082381

**Published:** 2024-04-09

**Authors:** Asif Umer, Mushtaq Ali, Ali Imran Jehangiri, Muhammad Bilal, Junaid Shuja

**Affiliations:** 1 Department of Computer Science & Information Technology, Hazara University, Mansehra 21130, Pakistan; ali_imran@hu.edu.pk; 2 School of Computing and Communications, Lancaster University, Bailrigg LA1 4YW, UK; m.bilal@ieee.org; 3 Department of Computer and Information Sciences, Universiti Teknologi PETRONAS, Seri Iskandar 32610, Malaysia

**Keywords:** analytical hierarchy process (AHP), computation-intensive tasks, delay-sensitive tasks, energy consumption, IoT task offloading & scheduling, smart transportation of logistics, task-aware scheduler, fault-tolerant manager

## Abstract

IoT-based smart transportation monitors vehicles, cargo, and driver statuses for safe movement. Due to the limited computational capabilities of the sensors, the IoT devices require powerful remote servers to execute their tasks, and this phenomenon is called task offloading. Researchers have developed efficient task offloading and scheduling mechanisms for IoT devices to reduce energy consumption and response time. However, most research has not considered fault-tolerance-based job allocation for IoT logistics trucks, task and data-aware scheduling, priority-based task offloading, or multiple-parameter-based fog node selection. To overcome the limitations, we proposed a Multi-Objective Task-Aware Offloading and Scheduling Framework for IoT Logistics (MT-OSF). The proposed model prioritizes the tasks into delay-sensitive and computation-intensive tasks using a priority-based offloader and forwards the two lists to the Task-Aware Scheduler (TAS) for further processing on fog and cloud nodes. The Task-Aware Scheduler (TAS) uses a multi-criterion decision-making process, i.e., the analytical hierarchy process (AHP), to calculate the fog nodes’ priority for task allocation and scheduling. The AHP decides the fog nodes’ priority based on node energy, bandwidth, RAM, and MIPS power. Similarly, the TAS also calculates the shortest distance between the IoT-enabled vehicle and the fog node to which the IoT tasks are assigned for execution. A task-aware scheduler schedules delay-sensitive tasks on nearby fog nodes while allocating computation-intensive tasks to cloud data centers using the FCFS algorithm. Fault-tolerant manager is used to check task failure; if any task fails, the proposed system re-executes the tasks, and if any fog node fails, the proposed system allocates the tasks to another fog node to reduce the task failure ratio. The proposed model is simulated in iFogSim2 and demonstrates a 7% reduction in response time, 16% reduction in energy consumption, and 22% reduction in task failure ratio in comparison to Ant Colony Optimization and Round Robin.

## 1. Introduction

**Figure 1:**
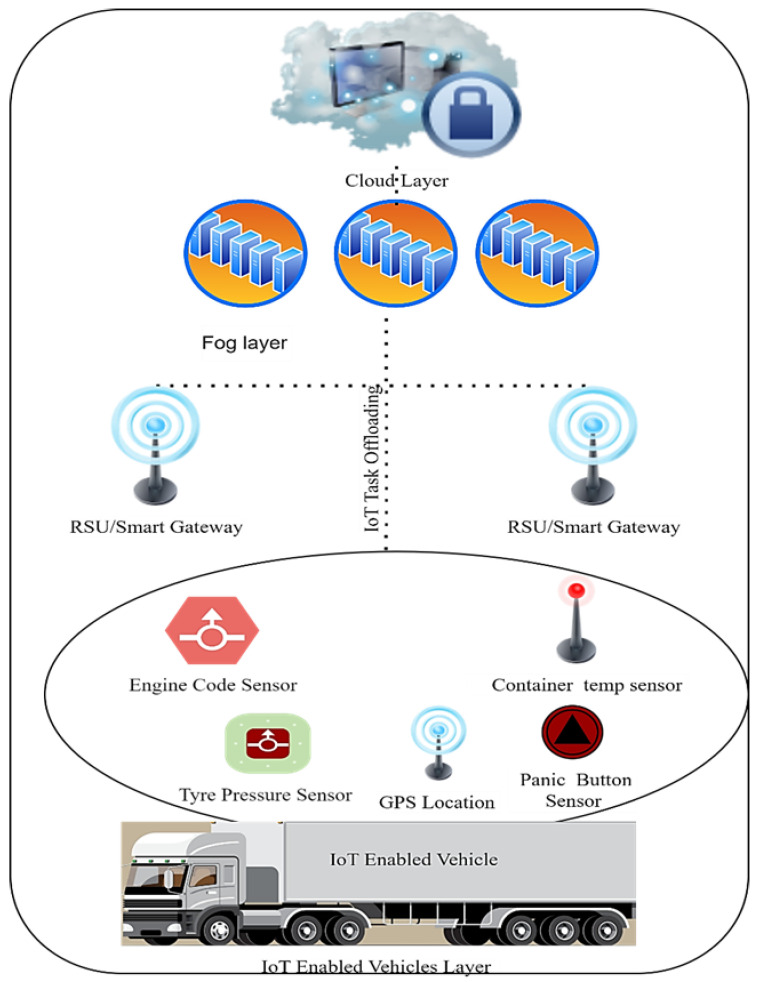
IoT-enabled vehicle.

The Internet of Things (IoT) is a setup of physical objects monitoring an environment using millions of sensor devices to produce large amounts of data and tasks. Due to limited battery power, storage, and processing capabilities, IoT tasks must be executed on remote servers to meet certain quality of service (QoS) requirements. Thousands of IoT devices (sensors) are connected to the internet for the purpose of communicating with servers and among themselves. However, there is a dire need for services that are near the IoT device and provide computation and storage facilities to the IoT devices and sensors [1]. One such scenario is for IoTs that monitor logistics. Figure 1 depicts the IoT logistics transportation vehicle information communication with logistic control and monitoring offices.

IoT devices and sensors often have storage, processing, and energy consumption shortcomings due to their small structures. To address these issues, IoT tasks and data are offloaded to the cloud and fog for service provisioning, reducing energy consumption and execution costs [2]. Cloud is one of the best solutions to storing and executing IoT device data. However, the time delay impedes the use of the cloud in delay-sensitive applications [3]. In this research, IoT-enabled vehicles are the focus, as they enable the efficient and reliable delivery of goods and materials. The IoT devices in these vehicles generate various types of data, ensuring a smooth and efficient journey for goods and vehicles. The IoT logistic vehicles generate a huge amount of data while monitoring the fleet on a real-time basis using sensors.

For this, a combination of cloud and fog can be used to meet the QoS requirements of the IoT devices. Cloud computing is a distributed model that is used for heavy computation and storage. But due to the distant nature of the cloud, high response times are experienced when offloading IoT devices. IoT devices can bring fog computing into service to bring cloud services nearby to the edge of the network. Fog nodes remove the limitations of cloud services, such as high response times [4,5]. IoT devices require high processing, energy, and storage capabilities due to their small sensors. To meet QoS requirements, they need to execute tasks on remote servers. To improve traffic flow and expedite freight movement, IoT logistics need efficient task offloading and scheduling strategies [6]. With ten-billion connected IoT devices installed and expected to grow to twenty-two billion in the next five years, this research aims to develop an efficient IoT logistics task offloading and scheduling model to reduce energy, task failure ratio, and response time [7].

To make the logistics process smooth and to monitor the fleet of organizations in real time, we need IoT sensors to be placed in vehicles to make the freight move smoothly. For this purpose, many companies are using IoT devices in logistics transportation to monitor the delivery of goods on a real-time basis, and by doing so, client satisfaction levels can be increased. Recently, IoT logistics have captured the attention of researchers, and billions of devices are expected to be installed in vehicles in the future. So, the IoT sensors have fewer computational resources to execute the tasks locally. Many IoT devices need to offload tasks to compute-in-reach servers, such as GPS sensors, tire pressure sensors, temperature sensors, front cameras, back cameras, etc. Front and back cameras of the IoT-enabled vehicles need image- and machine learning-based algorithms to process the data. Due to the limited computation resources of the sensors, we need to offload the tasks to fog and clouds to meet certain QoS requirements for the results [8].

A review of the previous work [1,2] revealed a multi-objective solution that caters to the following: (a) offloading and scheduling tasks based on priorities to decrease response time and energy utilization; (b) multiple parameter-based fog node selection, such as MIPS, BW, RAM, and node energy, to reduce energy footprint; (c) finding adjacent fog nodes based on task- and data-aware scheduling; and (d) IoT logistics truck requirement for task allocation with fault tolerance that has not been proposed. The proposed MT-OSF tackles IoT logistics challenges by assigning priorities to tasks and offloading them to coordinated cloud and fog nodes. Fog node allocation is based on parameters like energy, MIPS power, RAM, and bandwidth. The framework checks task execution and fog node failure while assigning tasks to nearby fog nodes to reduce the failure ratio.

**The following are the main contributions of the research:**

✓ IoT-enabled logistics vehicle tasks are classified as delay-sensitive and computation-intensive using the MT-OSF priority-based offloader to execute the important task on a priority basis on nearby fog nodes. Computation-intensive tasks are executed on cloud nodes using the FCFS algorithm.
✓ The MT-OSF Task-Aware Scheduler is proposed to allocate the offloaded tasks to the most efficient fog node. The analytical hierarchy process (AHP) is used to prioritize the most efficient fog node for IoT task execution and scheduling while considering RAM, BW, MIPS, and node energy.
✓ The Euclidean formula is used to calculate the shortest distance between the fog node and the IoT-enabled vehicle to whom the tasks are allocated for execution to reduce response time. Fault-tolerant manager is used and followed by task retry and node transfer mechanisms in case of task failure and fog node failure in the MT-OSF Task-Aware Scheduler to reduce the task failure ratio.
✓ By comparing the proposed MT-OSF with other standard algorithms, including Ant Colony Optimization and Round Robin, etc., we evaluated the system performance of the proposed MT-OSF. The suggested approach decreased the task failure ratio by 22%, reaction time by 7%, and energy usage by 16%.

The remaining paper is divided into several components. The related work is explained in Section 2 of the work, and the system architecture and proposed solution are presented in Section 3 of the paper. Section 4 presents simulations and results, while Section 5 presents the paper’s conclusion.

## 2. Related Work

IoT task offloading and scheduling enable the remote execution of tasks on servers to meet QoS requirements in smart transportation. Real-time monitoring of vehicles, cargo, and drivers enhances efficiency, reduces costs, and prevents cargo loss. IoT devices improve supply chain processes by enabling fleet tracking in real-time [9]. A vehicle monitoring system was developed to track vehicle travel from anywhere using GPS and GSM/GPRS technologies. The system uses GPS to obtain regular geographic coordinates, while GSM/GPRS updates vehicle location in a database. A smartphone app was also developed to continuously track the car’s whereabouts [10].

A car-locating system combining RFID, GPS, and GSM technologies was presented, enabling autonomous vehicle tracking without GPS signals. The project uses an RFID transmitter with a read range of 31 cm in the LF communication band. The system uses Bluetooth technology to transmit traffic status and density, allowing for effective data processing to determine dynamic traffic circumstances [11,12]. The article suggests developing 5G-based logistics models, combining 5G, IoT, and AI to create an intelligent traceability system for automated transportation. An IoT-based freight tracking system utilizes middleware, AI, RFID, GIS, and 3G connectivity, enabling real-time signal capture, data transfer, and information processing. Additionally, an IoT and RFID-based dynamic road transport monitoring system is shown [13,14,15].

EURIDICE is a freight solution that uses IoT technology to collaborate with highway infrastructure and databases. Without requiring human interaction, it provides cargo localization, rerouting, and monitoring of cargo conditions. The system also enables driver monitoring to identify healthy and unsafe driving behaviors. Wearable sensor networks enable low-power healthcare, real-time processing, and Internet of Things applications, reducing road accidents and enabling preventative actions. The IoT and fog-based computing system monitors driver behavior using sensors and communication technologies like RFID, Bluetooth, Wi-Fi, and 4G-LTE, enabling real-time monitoring and analysis. [16,17,18,19].

A driving style assessment solution was developed using an IoT-based embedded system that evaluates driving behavior based on factors like speed, acceleration, jerk, engine rotation speed, and driving duration. A monitoring system for IoT is crucial for smart logistics transportation, utilizing sensors to gather status data and pre-process them locally. Establishing a link with a logistics center is essential for communication and scheduling work. Wireless communication’s transmission rate and dynamic stability are crucial for real-time and stable information transmission in smart logistics transportation [20,21,22].

In [23], the authors proposed a LiMPO-based machine learning technique to offload mobile tasks to nearby edge nodes while considering the mobility of the users. Similarly, in [24], the authors proposed a deep learning-based energy-aware task-offloading scheme for 5G-enabled IoT devices. They did not use fault tolerance for task offloading and did not consider response time for their proposed algorithm. In [25], the authors proposed a content cache approach to enhance the performance of a proactive edge caching scheme based on federated learning (MPCF) while considering users preferences in edge computing.

In [26,27,28] the authors discussed and provided the task offloading schemes with limitations, similarly the other authors proposed secure task offloading scheme for drone technologies to gather and control the drone technology in efficient manner. However they emphasis that IoT task offloading and scheduling is still NP-Hard problem which need further analysis and require more optimize solution.

In [29], the proposed approach enhanced particle swarm fitness evaluation for load balancing, improving search efficiency and convergence speed. The multi-swarm design minimized local optimality. Experimental verification using Alibaba’s task dataset and benchmark algorithms demonstrate improved task scheduling performance in supply chain management. Similarly, in [30], the authors provide an elite-preserving genetic algorithm (ETS_GA)-based blockchain-assisted safe ES placement technique. Using niche sharing and tabu search, it addresses the classic GA’s premature issue. The algorithm employs blockchain-based privacy protection techniques and is continuously monitored. In [31] the authors present an osmotic approach for task offloading and scheduling, utilizing classifications of devices and tasks, and assigning tasks to suitable devices based on capacity. Compared to traditional random and Round Robin algorithms, the proposed algorithm outperforms others.

As discussed in the literature until now, researchers have mainly focused on IoT logistics structure, while in our research we basically focused on IoT smart logistics transportation task offloading and scheduling. So, in [32], the authors proposed a hybrid genetic-simulated IoT–Fog model while minimizing response time. They minimized the service delay; however, communication costs were not considered when calculating response time.

Swarm optimization and Ant Colony Optimization (ACO) methods were applied in [1] to effectively offload IoT chores to cloud and fog nodes. This reduced response time, but they did not apply any fault-tolerance mechanisms because most processes in ACO choose the shortest path, which increases the likelihood that the fog node will fail. In [33], a plan for IoT–mobile edge computing job offloading services is put out. This minimized energy consumption while considering computation and communication costs. However, they did not use the shortest path selection algorithm.

The author of [34] suggested a Dynamic Energy-Efficient Resource Allocation (DEER) load balancing approach to balance IoT workloads across fog nodes while using less energy. The scheduling of IoT tasks on fog nodes uses less energy. Fog node selection is based solely on a single metric; node energy, MIPS, RAM, or BW were not considered. To offload IoT tasks to fog nodes, the authors in [35] suggest an SDN-based path selection technique. By choosing the best fog nodes, they were able to shorten response times, although single-node failure might hurt the offloading procedure.

In [36], a hybrid model is provided by the authors in which they offload IoT tasks to fog or cloud nodes based on requirements using Q-learning algorithms. This efficiently balanced load on the fog nodes, but the model was limited to load balancing on just fog nodes. Response time was not considered. The authors proposed an IoT logistics monitoring system using mobile systems for vehicle temperature and humidity monitoring. However, fault-tolerance- and priority-based task offloading were not utilized. They focused on energy consumption and response time optimization, using the shortest path for delay-sensitive tasks. A fault-tolerance mechanism is needed for efficient models [37,38,39,40].

As analyzed from the literature review, IoT sensor nodes have limited battery life, storage, and computational power. IoT tasks need to be executed on powerful remote servers to extract information from the data generated from sensors. IoT tasks on remote servers are essential for meeting QoS parameters like connectivity, reliability, capacity, and latency. To reduce energy consumption and response time, researchers have developed fog computing to bring servers to near the network edge. Fog computing offloads and schedules tasks to fog and clouds, reducing energy consumption by prioritizing important and unimportant tasks and data on nearby fog nodes. Table 1 is used for a comparison of the different related work provided here in the paper.

IoT task placement based on single parameters, such as RAM usage, can increase response time and energy consumption. The Ant Colony Optimization (ACO) model for task offloading, which follows the shortest path after several iterations, can lead to increased task failure ratio and energy consumption. To improve offloading and scheduling, a framework should be provided to offload and balance IoT tasks, reducing energy consumption, response time, and task failure ratio. IoT task offloading and scheduling are NP-hard problems [1,2]. It has been analyzed from the literature that no one has used priority-basis task offloading, muti-criteria-based fog node selection, and fault-tolerant-based scheduling in IoT logistics.

IoT task offloading and scheduling have various challenges and issues that need to be addressed for an efficient IoT-based system. In task offloading, the offloading decision is critical in determining which data or tasks should be offloaded and which should be executed locally. In this regard, as from the literature review, various models are provided, but the problem is NP-hard, so we need an efficient solution for task offloading to reduce the energy consumption and computation costs as well. Secondly, in task offloading, most of the time similar tasks or data are offloaded to fog and clouds for execution, which increases energy consumption and response time. So, in the proposed work, we categorized the data into delay-sensitive and computation-intensive tasks/data to reduce energy consumption and response time. It was also observed in the literature review that some tasks or data do not offload and fail, so fault-tolerance-based task offloading is necessary to make the task offloading process or system efficient.

Once the tasks and data are offloaded to the nearby fog or cloud nodes, we need efficient scheduling techniques to utilize the resources efficiently. According to the literature review, multi-criteria-based fog node priority is not considered in scheduling, which results in fog node failure, a longer execution time, increased energy consumption, and an increased response time of the resources to the IoT devices. To consider and address all of the above challenges and limitations of IoT task offloading and scheduling, we need a framework that efficiently offloads the tasks to fog and clouds and schedules the offloaded tasks in a timely manner to reduce energy consumption and response time. So, we proposed Multi Objective Task-Aware Offloading and Scheduling Framework for IoT Logistics.

## 3. Proposed Work

In this section, we provide the system architecture, proposed MT-OSF model, entity interaction diagram, and time complexity of the proposed research work.

### 3.1. System Architecture

For IoT logistics, we proposed the Multi-Objective Task-Aware Offloading and Scheduling Framework (MT-OSF). The location of the logistic trucks, their temperature, and the interior state of the vehicle are all considered in the suggested model. Our centralized monitoring system will alert the administration and the affected cars if the IoT device data are discovered to be irregular. Task Manager is a smart gateway for gathering IoT tasks and sending them to cloud and fog nodes for further processing. The IoT Task Manager priorities jobs and data that are computationally and delay sensitive. The Task Manager will provide the Task-Aware Scheduler with lists of computation- and delay-intensive tasks, and the Task-Aware Scheduler will use the Euclidean formula to determine the distances between IoT devices and fog nodes, saving the results in the repository. The analytical hierarchy process (AHP) is used to determine the cumulative weight of fog nodes, and the priority vector is then saved in the repository. Offloaded tasks are placed on nearby fog nodes and cloud nodes according to the priority repository information. The fault-tolerance manager verifies the successful allocation of tasks after assigning IoT tasks to fog or cloud nodes. If any tasks fail to be allocated on the execution machine, the fault-tolerance manager will reallocate them to another active node.

### 3.2. Proposed Work/MT-OSF

We propose the Multi-Objective Task-Aware Offloading and Scheduling Framework (MT-OSF) for IoT logistics. In the proposed model, we obtain tasks from the vehicles that have IoT devices. We have V_1_, V_2_, V_3_, …, V_n_ logistics vehicles that have IoT devices. For simplicity, we named the IoT devices which are placed at vehicles as VIoT_1_, VIoT_2_, VIoT_3_, …, VIoT_m_. We have multiple smart gateways (SG_1_, SG_2_, and SG_k_) to receive IoT tasks, calculate the shortest path, and prioritize the task into computation-intensive and delay-sensitive categories. We have fog and cloud nodes for the execution of delay-sensitive tasks and computation-intensive tasks. The fog nodes are Fog_1_, Fog_2_, Fog_3_, …, Fog_z_ and the cloud nodes are Cloud_1_, Cloud_2_, Cloud_3_, …, Cloud_x_. Furthermore, each fog node has multiple Virtual Machines (VMs) such as VM_1_, VM_2_, …, VM_t_. The tasks that are generated by IoT devices are considered Task_1_, Task_2_, Task_3_, …, Task_s_. We have used a random model (Poisson process) for IoT task generation, such as that any vehicle can ask for the total remaining distance from his destination, and temperature measurements are sent to the admin office for further necessary action and orders. The tasks that are generated by the IoT devices (1, 2, 3, …, m) by the specific vehicle (1, 2, 3, …, n) are represented as the tasks generated by the IoT devices that are received by a smart gateway and prioritized as delay-sensitive or computation-intensive tasks. Delay-sensitive tasks are those tasks that need immediate execution and action within the required time, otherwise, the important information will be lost.

The computation required by the IoT task and the time for the completion of the task will be evaluated, and the total weight (M) is calculated for the generated tasks using Equation (1) as follows:

(1) $$M_{n,s,m}=\sum_{s=1}^{i}{Service\_Time}_{s}/{time}_{s}$$

If the values of M of the concerned task, concerned vehicle, and concerned IoT device are >0.5, then such tasks will be put into a delay-sensitive queue and forwarded to a task-aware scheduler for further allocation to a nearby fog node. Computation-intensive tasks are those that need execution for a long time. Tasks generated by IoT devices with computation required and time for completion are received by the IoT Task Manager, which calculates the total weight (M) of the tasks according to Equation (1).

**Figure 2:**
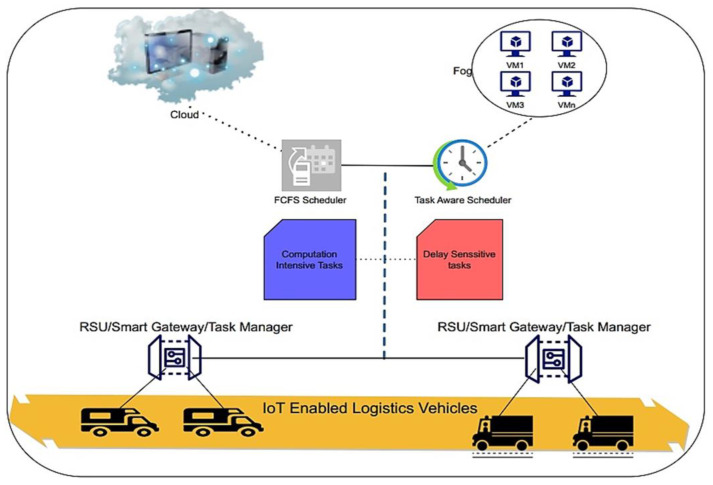
IoT Task Manager (Smart gateway).

If the value of M is ≤ 0.5 then such tasks are put to a computation-intensive queue. Delay-sensitive tasks are offloaded to fog nodes while computation-intensive tasks are offloaded to cloud nodes. Figure 2 is used to show the process of algorithm one. Table 2 is used to show the abbreviations and their meaning of the used terms and symbols in the proposed research work.

The IoT tasks created by the devices at the smart gateways are received using Algorithm 1. The concerned vehicle’s IoT device duties are sent to the smart gateway. Equations (1) and (2) are used to compute the task’s overall weight.
**Algorithm 1.** IoT task offloading and task categorization as delay-sensitive and computation-intensive based on required resources and deadline.
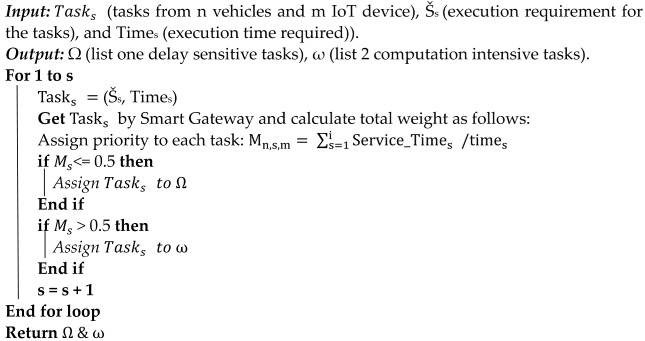


Tasks are allocated to computation-intensive list if the sum of their weights higher than 0.5, and delay-sensitive jobs if the sum is less or equal to 0.5.

The two lists of jobs are sent to the Task-Aware Scheduler (TAS), which functions on a fog main node once tasks have been successfully prioritized. When using a task scheduler, computation-intensive work is sent to cloud nodes while delay-sensitive work is sent to nearby fog nodes. The analytical hierarchy process (AHP) is used to examine and compare the performance of the available fog nodes. One method for making decisions using several criteria is the analytical hierarchy process (AHP). From paired comparisons, ratio scales are derived using this technique. Measurements like cost, weight, and other factors may be used as input, as well as objective evaluations like satisfaction, emotions, and preferences.

**Figure 3:**
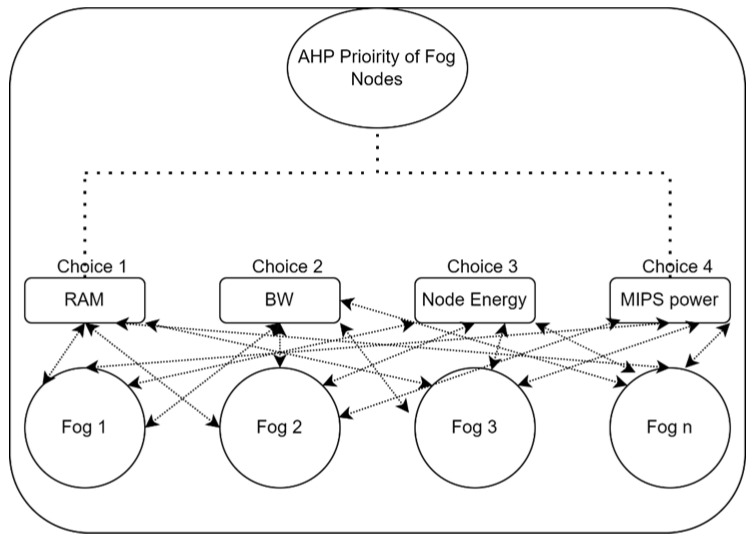
AHP for fog priorities calculation.

AHP allows for a little level of judgement error since humans are not always consistent. The significant Eigenvalue is used to generate the ratio scales, whereas the significant Eigenvalue is used to calculate the consistency index. Figure 3 shows the AHP for ranking fog nodes according to priority. For IoT logistics tasks, we select the top fog node from a range of options to perform them successfully and on time. Each fog node is composed of several Virtual Machines (VMs). It is used to choose the fog node based on the top VM that is currently housed there. For instance, if of the five fog nodes, fog node 2 has the best available VM, we will assign and prioritize that fog node to complete IoT activities. From among the several fog nodes (FNs) that we have, we must choose the right one. Each fog has unique VMs, and each VM also has unique processing power, RAM, and bandwidth requirements, as well as node energy requirements.

To locate the appropriate VM that satisfies the user’s criteria, the configuration of each accessible parameter of the VM is compared with the setting of the required parameter of the VM. The proposed method uses the AHP procedure to create a Matrix with the necessary value. RAM, MIPS processing power, bandwidth, and node energy were the four factors we used. Thus, in terms of the AHP method, we need a 4-by-4 matrix. The values for the diagonals are represented as 1, and the values for the chosen choice are written above the diagonals. The values are expressed in a reciprocal form in accordance with the AHP procedure if the choice value is on the left side of the comparison line and the chosen option is on the right side of the middle point.

Equation (3) was used to enter the matrix’s top diagonal values. We utilized a range of 9, 7, 5, 3, 1, 3, 5, and 7. We placed the parameters of the fog node, such as RAM, MIPS, bandwidth, and node energy (NE), on the left and right sides of the decision values. Equation (3) was used to calculate the wagon vector matrix values from the choice values. $a_{ij\;}$ was used to specify the row- and column-wise values of the AHP matrix, which was further solved and retrieved the priority values of the fog nodes. The values on the right side of the middle values from the submitted choices were put to the matrix in the same form using Equation (3) while the choice values on the left side of the middle zero value were put into the matrix below the diagonal in reciprocal form using Equation (4).

Equations (2)–(8) are used to obtain the priority values by using the AHP. First, the fog node input choice values are obtained from the fog nodes, such as RAM, MIPS, bandwidth, and node energy. First, we obtained all the values for the AHP matrix one by one by using Equations (2) and (3), and then, by using Equations (4)–(8), the proposed framework calculated the priority values of the fog nodes while considering the VMs of the nodes. Once the priority list of the fog nodes was finalized, we assigned the top-most task to the top-most fog node to reduce the energy consumption, execution cost, response time, and overall efficiency of the proposed system.

(2) $$AHP\_Matrix\_Diagnol\_Right=a_{ij\;}$$

Similarly, the lower diagonal values were put into Equation (3) using the upper diagonal values that were put into the matrix by using Equation (2). The i and j are the rows and columns of the matrix. Equation (2) was used to calculate the wagon vector matrix values from the choice values. The equation was used to specify row and column wise values of the AHP matrix, which was further solved and retrieved the priority values of the fog nodes. Equation (2) is the part of AHP where we need to specify or obtain one value for matrix development. By obtaining all the values using Equation (2), the final matrix was built for further wagon vector values/priority values calculation.

(3) $$AHP\_Matrix\_Diagnol\_Left=\frac{1}{a_{ij}}$$

From the matrix that is constructed by using Equations (2) and (3), we further calculated the weight (W) of each fog node individually using Equations (4)–(7) and calculated the cumulated weight of the fog nodes using the total power, total RAM, total energy, and total bandwidth of the node using Equation (9).

(4) $$W1=\sum_{z=1}^{z}\sum_{t=1}^{t}MIPS$$

(5) $$W2=\sum_{z=1}^{z}\sum_{t=1}^{t}RAM$$

(6) $$W3=\sum_{z=1}^{z}\sum_{t=1}^{t}BW$$

(7) $$W4=\sum_{z=1}^{z}\sum_{t=1}^{t}NE$$

(8) $$Total\_W=\sum_{z=1}^{z}\left(W1+W2+W3+W4\right)$$

Equation (4) was used to calculate the weight of a fog node VM in the context of MIPS power; Equation (5) was used to calculate the RAM power of a fog node VM; Equation (6) was used to calculate the bandwidth of the fog node; Equation (7) was used to calculate the weight of a fog node energy (NE); and Equation (8) was used to calculate the total weight of a fog node. The fog nodes with high weights are kept at the top of the priority list, and so on.

To schedule the IoT tasks in a way that might save energy and lower the task failure ratio, the Task-Aware Scheduler (TAS) first determines the priority of the fog nodes. To speed up reaction times, the TAS also determines the shortest route between IoT-enabled cars and fog nodes. Equation (9) was utilized to determine the route between the IoT-enabled logistic vehicle and the Fog node.

(9) $${\;D}_{z}^{n}=sqrt\{\left(xm–xn\right)2+\left(ym–yn\right)2\}$$

**Figure 4:**
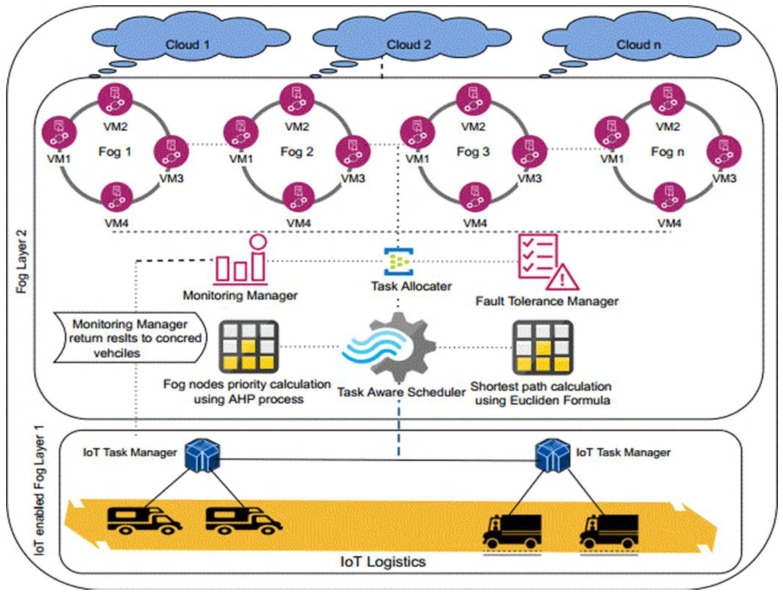
MT-OSF Task-Aware Scheduler.

In the equation, we used the Euclidean formula, and z and m represent the IoT-enabled vehicle and the fog nodes. Figure 4 is used to represent the working of the Task-Aware Scheduler. After the cumulative weight calculations, the list of the fog node is provided in ascending order as if any fog node has low cumulative weight, and then our proposed algorithm will give priority to that fog node.

The Task-Aware Scheduler uses the AHP method to determine the priority of fog nodes and the Euclidean Formula to determine the shortest path between fog nodes and IoT devices.

According to the data that are kept in the scheduler repositories, the Task Allocator Module distributes delay-sensitive jobs to the closest fog nodes. Tasks that need a lot of computation are offloaded to the cloud. If jobs are successfully placed on the execution machine, the fault-tolerant manager examines and validates this; otherwise, alternative re-allocation procedures are employed.

Fog and cloud nodes carry out the tasks, and the outcomes are communicated back to the source for any additional action that may be required. Using IoT devices, the suggested strategy essentially allows for efficient fleet management of logistics. The MT-OSF Algorithm 2 is used to generate the matrix of the necessary options after obtaining priority values from IoT tasks. The two lists created by Algorithm 1 serve as the input for Algorithm 2. The algorithm’s output is the priority list for fog nodes and the quickest route for logistics trucks to arrive to the fog nodes.
**Algorithm 2.** Fault-tolerance-based IoT tasks scheduling on fog nodes using a multi-criterion decision-making process (AHP) and shortest distance calculation using the Euclidean formula.
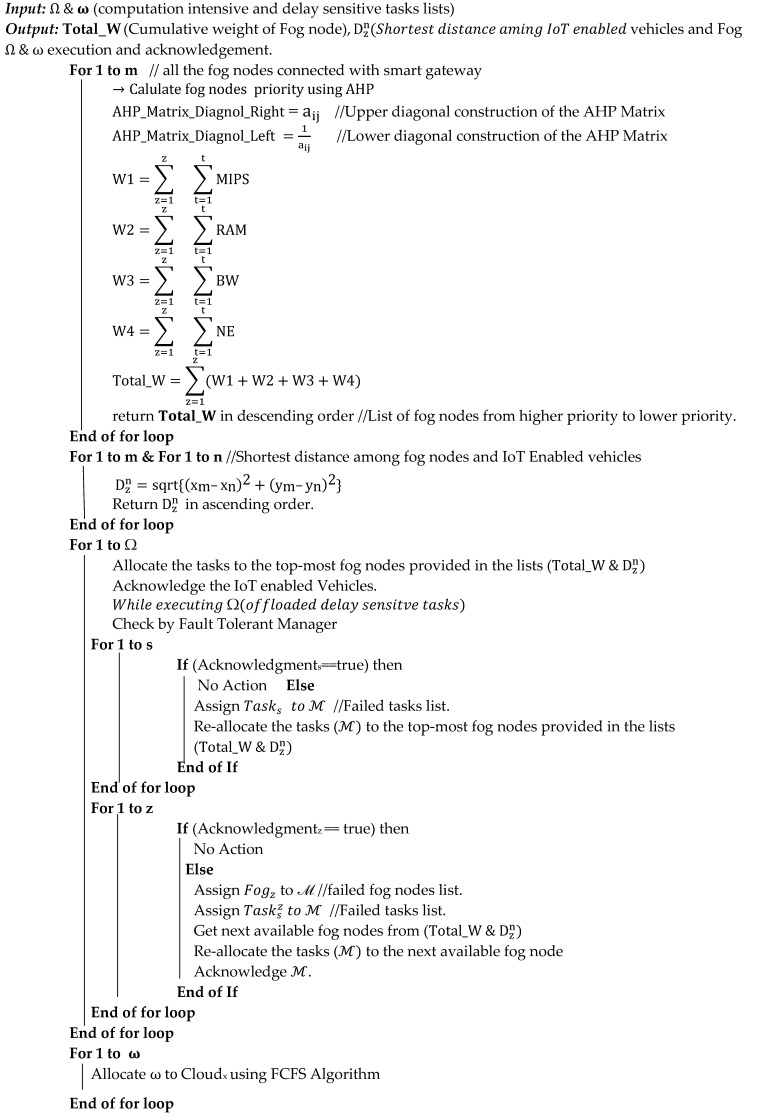


The system distributes the IoT logistics truck duties to the adjacent fog nodes with efficient node energy, RAM, bandwidth, and MIPS power after determining the shortest distance and fog node priority. Algorithm 2 distributes and arranges IoT jobs on fog nodes to minimize energy consumption and response time. Fault tolerance is an important factor in resource allocation, as when we allocate a task to a fog node, if the task fails and does not execute due to some reason, then important data or tasks may not be able to perform the work. So, in the proposed model, we used a fault-tolerance manager to check and verify successful task allocation and execution to fog nodes from IoT devices.

In MT-OSF Algorithm 2, the fault-tolerant manager will be launched if either the node to which the tasks are assigned fails or the tasks (which are allocated to the required resources) are unable to be completed. The “Fault-tolerant Manager” component handles “Task-based fault-tolerance” and “node-based fault-tolerance”, two key fault-tolerant procedures. First, if a failed task occurs at any node, “Task-based fault-tolerance” re-executes it. The work will be moved to another nearby fog node if one fog node fails.

## 4. Simulation Setup and Results

In a real-world environment of IoT-enabled logistics vehicles, it is difficult to test and check the proposed algorithm in a repeatable and controllable manner. So, we used a simulation/implementation-based strategy to test and check the proposed “Multi-Objective Task-Aware Offloading and Scheduling Framework for IoT Logistics (MT-OSF)” model. We used FogSim2, the latest simulation, which is used for IoT task offloading and scheduling in cloud and fog computing. The details of the simulation setup are given below.

### 4.1. Resource Modelling

iFogSim2 [41] was used to implement and simulate the proposed model. Different tasks and data were created in Java-based classes while offloading and scheduling them on fog datacenters and cloud datacenters. The tasks were generated randomly with the required resources, such as MIPS, bandwidth, RAM, and time for completion of the task. For the simulation of the proposed model, we used a Core i3 system with 8 GB of RAM and a 500 GB hard drive to run and store the IoT tasks. Table 3 shows the parameters used in the simulation with the given values.

### 4.2. Evaluation Parameters

For performance evaluation, we used the following parameters: energy consumption, response time, and task failure ratio. These are necessary parameters for the evaluation of the results of the proposed research.

#### 4.2.1. Response Time

Response time is the type of QoS that will be checked for IoT tasks and data. The response time of the IoT tasks can be calculated by the following equation, in which response time (RT) is the sum of the IoT–fog communication cost (IFC), fog–cloud communication cost (FCC), and average service time (AST) on fog nodes.

(10) RT=IFC+FCC+AST

#### 4.2.2. Energy Consumption

The most important variable in determining how well the suggested model performs is energy consumption, since we aim to decrease fog node energy consumption while offloading IoT activities. We can lower energy use when the offloaded jobs are efficiently balanced. The real energy used during task submission and execution is the energy associated with computation and communication. We used and evaluated energy consumption according to the following equation:

(11) ℇc=ℇce+ℇcc

#### 4.2.3. Task Failure Ratio

Task failure ratio is the percentage in which the number of successful and failed tasks are given in different simulation runs.

(12) Tf=Number of Tasks Failed×100/total Number of Tasks

### 4.3. Results

In this section, we provide the results obtained from the simulation. We ran the simulation with different parameters of the values and obtained the most suitable results, which are given in the following scenarios:

#### 4.3.1. Scenario 1

**Figure 5:**
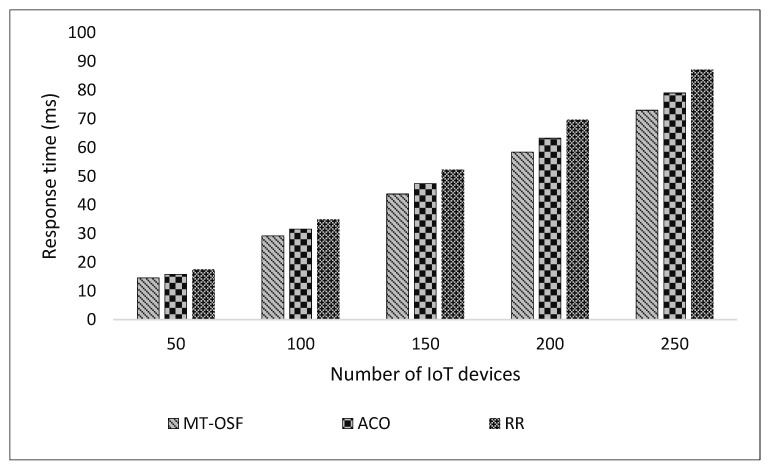
Response time of fog nodes.

We created one cloud data center, in which we created ten Virtual Machines and 500 MB of storage. Similarly, we created two fog nodes, each with four Virtual Machines. The simulation parameters in Table 3 were used to evaluate the response time and energy consumption of the proposed model. The proposed model was compared with the latest research work [1], and it was observed that the MT-OSF performed very well in terms of response time as the algorithms prioritized the tasks and then executed the important tasks on nearby fog nodes using the AHP process and Euclidean formula. Figure 5′s *x*-axis shows the number of internet devices attached to the vehicle, while the *y*-axis represents the response time in milliseconds. The proposed model was compared with Round Robin (RR) and Ant Colony Optimization (ACO) [1]. The results show that the proposed model, MT-OSF, performed well and reduced the response time from 87 milliseconds to 73 milliseconds. The response time is given in Figure 5 for delay-sensitive tasks that were executed on nearby fog nodes.

In Figure 5, we provide the response time of the important and delay-sensitive tasks that were executed on fog nodes.

**Figure 6:**
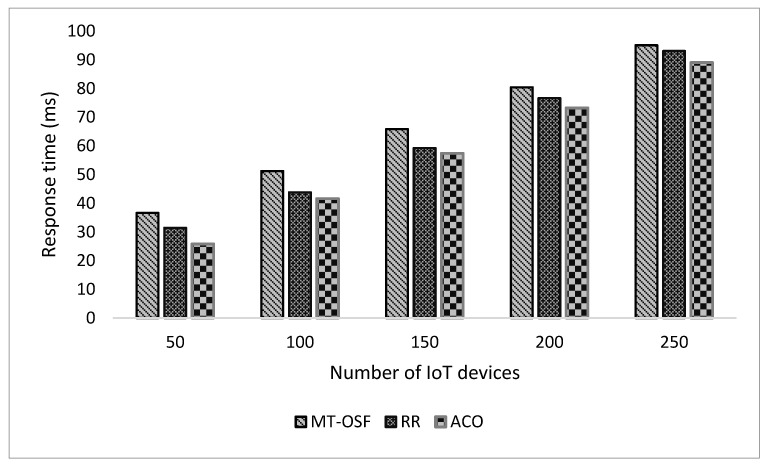
Response time comparison of computation-intensive tasks on cloud nodes.

In Figure 6, we provide the response times of the computation-intensive tasks and normal tasks that were executed on cloud nodes or cloud datacenters. Figure 5’s *x*-axis shows the number of internet devices attached to the vehicle, while on the *y*-axis, the figure represents the response time in milliseconds.

**Figure 7:**
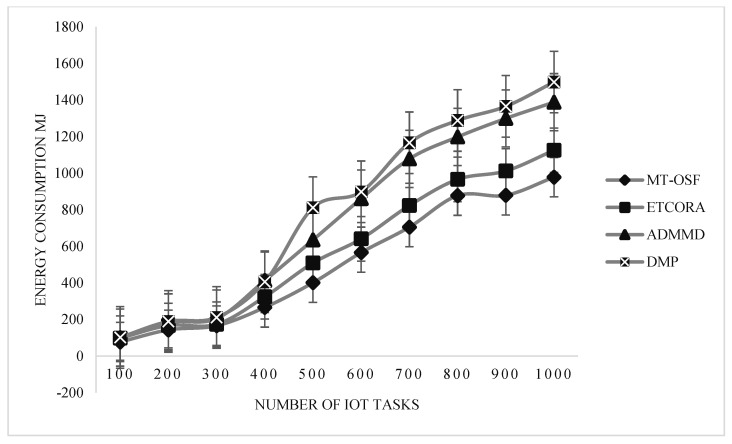
Energy consumption comparison of delay-sensitive tasks on fog node.

In Figure 7, an energy consumption comparison is provided for the delay-sensitive tasks. On the *x*-axis, the number of IoT tasks is provided, while on the *y*-axis, the energy consumption values are provided in megajoules.

**Figure 8:**
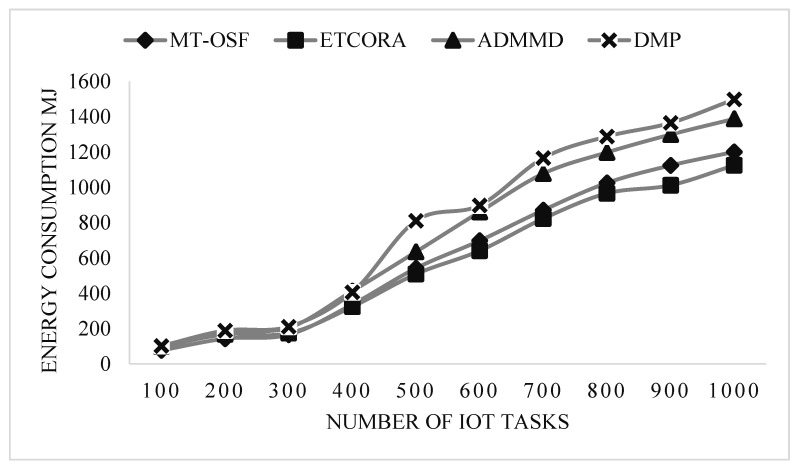
Energy consumption of computation-intensive tasks on cloud nodes.

Our proposed model employed the AHP for fog node priority and IoT task scheduling, as shown in Figure 7. It performed extremely well in terms of energy usage on the fog layer. These fog nodes are assigned to IoT tasks via the AHP, a multi-criteria decision-making mechanism that favors jobs with greater RAM, MIPS, bandwidth, and energy on the node. We decreased the cost of communication, and when the fog node was assigned to the work, it completed 90% of the mission’s needs. In terms of energy usage, the suggested model also contrasted with the most recent work for computation-intensive activities. Figure 8 is used to show the energy consumption comparison of the proposed model with ETCORA, ADMMD, and DMP.

In Figure 8, an energy computation comparison is provided for the computation-intensive tasks. On the *x*-axis, the number of IoT tasks is provided, while on the *y*-axis, the energy consumption values are provided in megajoules. From Figure 8, it is observed that the proposed model’s energy consumption increases due to an increase in communication costs.

The proposed model was compared with Round Robin (RR) and Ant Colony Optimization (ACO). The results show that the response time of the proposed model, MT-OSF, increases for computation-intensive tasks, while the other two techniques, such as RR and ACO, decrease the response time in comparison with the proposed model. For computation-intensive tasks, we used the First Come, First Serve (FCFS) basis scheduling algorithm, and executed it on a cloud data center. In scenario 1, we also evaluated the proposed model (MT-OSF) using Equation (12) for energy consumption. The proposed model for energy consumption is compared with the latest related work [39], such as ETCORA, ADMMD, and DMP.

**Figure 9:**
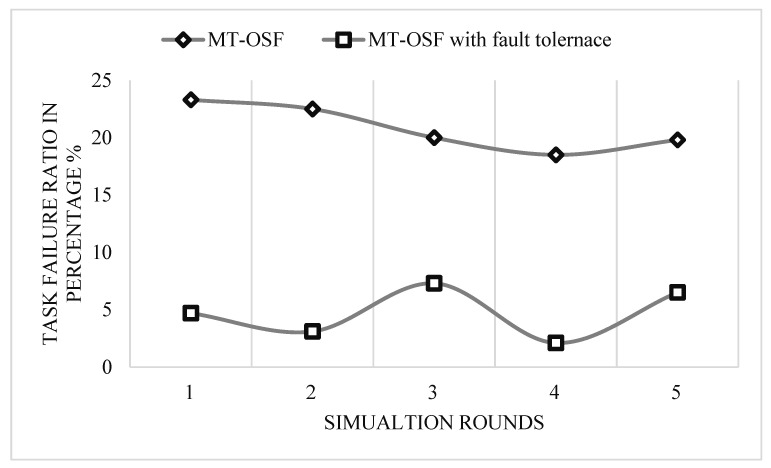
IoT task failure ratio with and without fault tolerance.

The proposed MT-OSF model did not use the shortest path algorithm, so in multiple cloud nodes it increases energy consumption and response time as we use the FCFS algorithm for scheduling. If we use the AHP for cloud VM priority calculation and scheduling, then we can decrease energy consumption and response time, but in this research, we only focus on IoT logistics task offloading and scheduling to fog nodes. Figure 9 presents a comparison of the MT-OSF with and without fault tolerance. From the results, it is analyzed that task failure ratio is decreased by 22% in comparison to without the fault-tolerance MT-OSF Algorithm 2.

#### 4.3.2. Scenario 2

**Figure 10:**
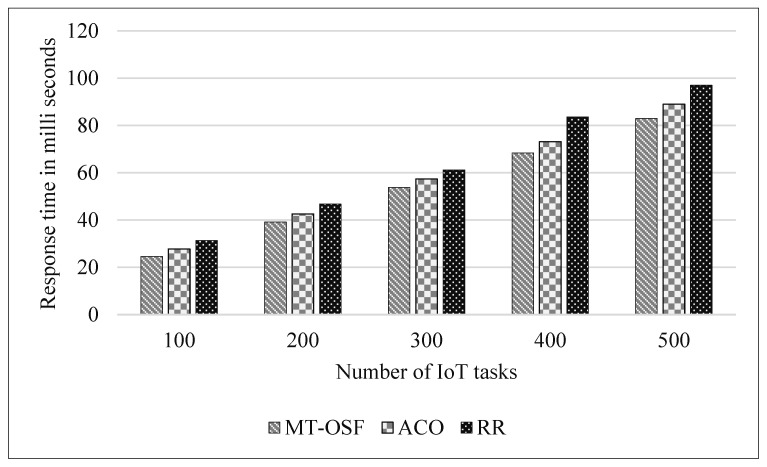
Response time comparison of the proposed model for delay-sensitive tasks.

In scenario two, we increased the fog nodes up to 10, and each fog node had four virtual machines with 50 MIPS of computational power, 10 MB of bandwidth, 2 MB of RAM, and 500 megajoules of energy. Five cloud nodes were created, each with ten VMs having computational powers such as 1000 MIPS, 50 MB of bandwidth, and 8 MB of RAM. The parameters remained the same as in scenario 1. We generated random tasks provided by the iFogSim2 task class, in which we first considered 100 tasks, then 200 tasks, and then up to 500 tasks for performance evaluation. In Figure 10, we provide the results of the comparison of the proposed model with the RR and ACO models. The proposed model reduced the response time for delay-sensitive tasks as we used the shortest path formula along with the AHP to schedule the tasks on nearby fog nodes. As is shown in Figure 10, the communication cost increases due to more cloud nodes and fog nodes, so with increased communication costs, the response time of the IoT tasks also increases by 10%.

**Figure 11:**
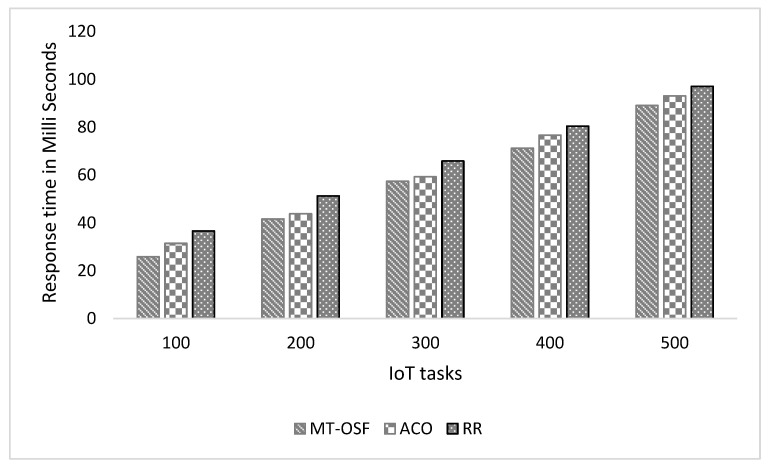
Response time comparison of the proposed model for computation-intensive tasks.

The proposed model performed very well as the AHP returned the fog nodes that had the computational power, RAM, BW, and node energy to execute the tasks. In the other methods, such as RER and ACO, few iterations were needed as they do not look for efficient fog nodes; they only see the near fog nodes and allocate tasks. Similarly, we used the AHP for the VMs of cloud nodes as well, and then we compared the proposed model with the RR and ACO models, where the proposed model also decreased the response time for computation-intensive tasks. Figure 11 is used to show the response time comparison of the proposed MT-OSF model with RR and ACO, which are the last offloading models.

**Figure 12:**
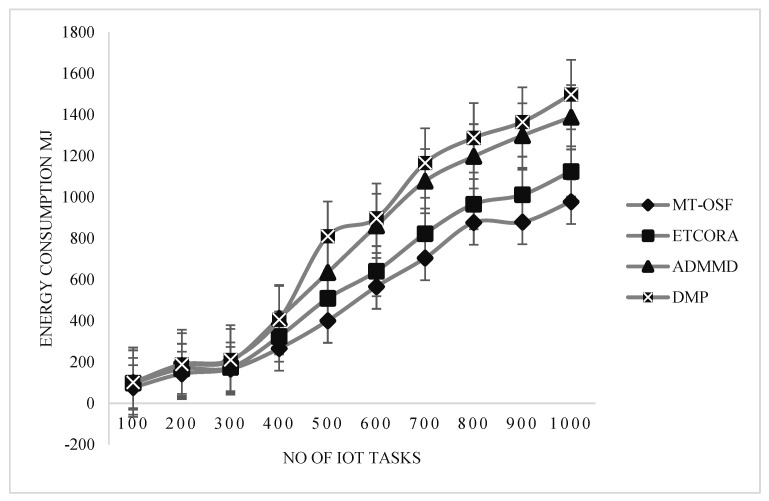
Energy consumption comparison of delay-sensitive tasks on fog nodes.

**Figure 13:**
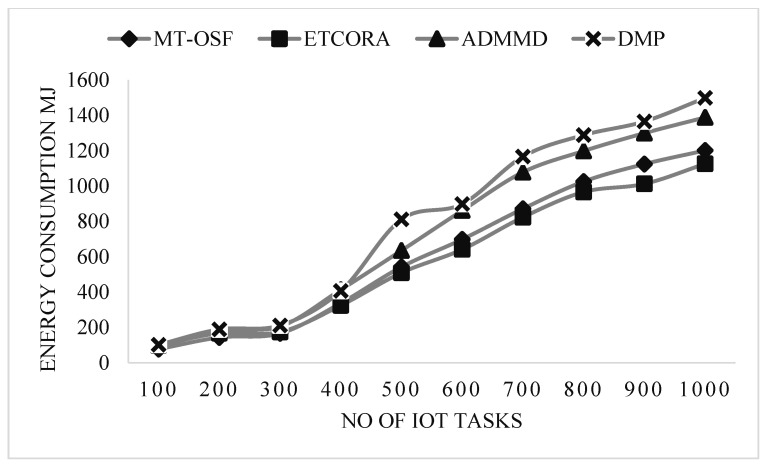
Energy consumption of computation-intensive tasks on cloud nodes.

In scenario 2, the proposed model was also evaluated for energy consumption, using Equation (12). In Figure 12 and Figure 13, it is observed that the proposed model decreased energy consumption in comparison with the latest available work in energy composition for IoT task offloading and scheduling. Overall, energy consumption increased by up to 5% when the fog and cloud nodes were increased, so greater communication cost was used due to priority calculation and other factors. In Figure 12 and Figure 13, energy consumption comparisons are provided for delay-sensitive tasks and computation-intensive tasks. On the *x*-axis, the number of IoT tasks is provided, while on the *y*-axis, the energy consumption values are provided in megajoules. From Figure 12 and Figure 13, it is observed that the proposed model’s energy consumption decreased as we used the proposed model for fog and cloud nodes. Thus, we reduced the communication cost and allocated the most efficient VMs on the cloud and the most efficient fog nodes on the fog layer to computation-intensive and delay-sensitive tasks.

## 5. Conclusions

This research focuses on IoT task offloading and scheduling models for logistics vehicles, specifically for mega projects like the China–Pakistan Economic Corridor. This study presents a Multi-Objective Task-Aware Offloading and Scheduling Framework for IoT Logistics (MT-OSF), which prioritizes tasks into delay-sensitive and computation-intensive tasks using a priority-based offloader. The TAS uses the analytical hierarchy process (AHP) to calculate the priority of fog nodes for task allocation and scheduling based on factors such as node energy, bandwidth, RAM, and MIPS power. The MT-OSF also calculates the shortest distance between the IoT-enabled vehicle and the fog node to which the IoT tasks are assigned. The system schedules delay-sensitive tasks on nearby fog nodes and allocates computation-intensive tasks to cloud data centers using the FCFS algorithm. The fault-tolerant manager checks task failure, re-executing tasks if necessary and allocating tasks to another fog node to reduce the task failure ratio. The MT-OSF reduced response time by 7%, energy consumption by up to 17%, and reduced task failure ratio by 22% in comparison to Ant Colony Optimization and Round Robin. Future research aims to use machine learning-based approaches to train the offloading model and develop a framework for executing delay-sensitive and computation-intensive tasks while predicting IoT mobility.

## Figures and Tables

**Table 1:** Literature review comparison.

Reference	Response Time	Task Priority	Energy Consumption	Fault Tolerance	Multi-Criteria-Based Fog Node Allocation	Simulation Tool Used
[1]	√	×	×	×	×	MATLAB-R2023b
[11]	√	×	×	×	×	Arduino based IoT real setup
[32]	√	×	×	×	×	iFogSim2
[33]	×	×	√	×	×	iFogSim
[34]	×	×	√	×	×	MATLAB
[35]	√	×	×	×	×	iFogSim
[36]	×	×	√	×	×	iFogSim
[37]	√	×	×	×	×	CloudSim 3.0.3
[38]	√	×	√	×	×	SIMUL8
[39]	√	×	√	×	×	iFogSim
[40]	√	√	√	×	×	C++ based NS3 Tool
MT-OSF Proposed Model	√	√	√	√	√	iFogSim2

**Table 2:** Abbreviations used in the proposed model.

Symbol	Abbreviation	Symbol	Abbreviation
*TAS*	Task-Aware Scheduler	${Task}_{n}^{m}$	Task generated by logistic vehicle IoT device
*SG*	Smart Gateway	*VM*	Virtual Machine
*VIoT*	IoT devices placed in vehicles	*V*	IoT-enabled vehicle
*ACO*	Ant Colony Optimization	*MIPS*	Million instruction per second
*PSO*	Particle swarm optimization	*BW*	Bandwidth
*RAM*	Random access memory	*IoT*	Internet of Things
*Ω*	Delay-sensitive tasks	*ω*	Computation-intensive tasks
*Š_s_*	Execution requirement for the tasks	*Time_s_*	Execution time required
*AHP*	Analytic hierarchy process	M	Weight of the IoT task
*NE*	Fog node energy	*FN*	Fog node
$D_{m}^{n}$	Shortest distance between fog node and IoT device	*W*	Weight of the fog node, BW, MIPS power, RAM, and node energy
*C.W*	Cumulative weight (combined weight of the four parameters)	*GPS*	Global positioning System
*N*	IoT-based vehicles	*S*	IoT tasks
*M*	IoT devices/sensors	*z*	No of fog nodes
*X*	No of cloud nodes	*T*	No of Virtual Machines

**Table 3:** Simulation parameters.

S. No	Simulation Parameters	Value	Description
1	Cloud_x_	1	One cloud data center created
2	${Fog}_{i}$	10	10 fog nodes created
3	Š_s_	5–10 MIPS	Each task processing requirement
4	Time_s_	2–6 ms	Each task required time for execution
5	Smart Gateways	2	Each gateway connected with 5 fog nodes
6	Logistics Vehicles	5	--
7	IoT devices/Sensors	50 × 5 = 250	Each vehicle has 50 sensers placed in it
8	BW	5–10 MHz	Bandwidth for communication lines
9	Processing Capabilities	50–100 MIPS & 500 to 1000 MIPS	Fog nodes and cloud nodes processing power
10	Task Size	250 kb–1 MB	--
11	Latency from IoT device to fog	2–20 ms	--
12	Latency from IoT device to cloud	30 ms	--

## Data Availability

Data are contained within the article.
